# Outpatient Antibiotic Prescribing for 357,390 Children With Otitis Media

**DOI:** 10.1097/INF.0000000000003693

**Published:** 2022-09-07

**Authors:** Péter Csonka, Sauli Palmu, Paula Heikkilä, Heini Huhtala, Matti Korppi

**Affiliations:** From the * Department of Pediatrics, Tampere University Hospital, Tampere University, Faculty of Medicine and Health Technology, Center for Child, Adolescent, and Maternal Health Research; † Terveystalo Healthcare, Tampere, Finland; ‡ Faculty of Social Sciences, Tampere University, Tampere, Finland

**Keywords:** otitis media, antibiotic prescribing, guideline concordance, outpatient, management

## Abstract

This study aimed to evaluate antibiotic prescriptions for children with otitis media (OM) in a large nationwide network of private clinics between 2014 and 2020. Special focus was paid to amoxicillin and amoxicillin-clavulanic acid prescriptions, which the national guidelines recommend for first-line drugs. We also focused on macrolides, which are commonly prescribed for respiratory tract infections in children but are not appropriate for OM cases.

**Methods:**

The data were obtained from the electronic health records of about 250,000 annual visits in <18 years old children. The collected variables were all diagnoses, age, visit year, doctor’s specialty, and prescribed antibiotics. Children with OM and upper respiratory tract infections were included, but those with lower respiratory tract infections were excluded.

**Results:**

The number of children with OM was 357,390 (55.2% boys). Antibiotics were prescribed in 44.8% of cases, with the lowest proportion (44.1%) being in <2 years old children. The overall prescription rate decreased from 48.3% in 2014 to 41.4% in 2020. The rates were 19.3% and 18.1% for amoxicillin and 12.6% and 13.2% for amoxicillin-clavulanic acid, respectively. Macrolide prescriptions were reduced from 7.5% to 3.5%. Pediatricians prescribed antibiotics for 38.8%, general practitioners for 54.0% and ear, nose and throat physicians for 39.8% of children with OM.

**Conclusion:**

The selection of antibiotics for OM, when prescribed, was according to the recommendations (amoxicillin or amoxicillin-clavulanic acid) in 80.1% of pediatricians, 67.0% of general practitioners and 55.1% of ear, nose and throat physicians.

Otitis media (OM) is a common complication of respiratory tract infections in children. Over 130,000 acute OM cases were diagnosed and treated in Finnish children in public health care centers in 2014.^1^ Nearly half of the infants have suffered from acute OM during their first living year and 70% before the age of 2 years.^1^ Among 17 European evidence-based guidelines, all recommend analgesics, and 15 recommend the wait-and-see practice in selected cases, and when treated, 14 recommend amoxicillin as the first-choice antibiotics: 7 with the dose of 40–50 mg/kg/day and 7 with the dose of 90 mg/kg/day.^2^

The Finnish Current Care Guidelines for diagnosis and treatment of OM, which were published in 2011 and updated in 2017, are in line with the European guidelines.^1^ The Finnish guidelines recommend that children younger than 24 months of age, and children with bulging eardrum, draining middle ear or purulent OM in both ears should be treated immediately, and the first-line antibiotics are amoxicillin or amoxicillin-clavulanic acid.^1^ High amoxicillin dosing is not needed due to the low frequency of pneumococcal penicillin resistance in the country. Second-generation cephalosporins, trimethoprim-sulfa and macrolides are the second-line drugs recommended for those allergic to penicillin. Children 2 years of age and upward with less prominent clinical findings can be left without antibiotics, but they must be reexamined after 2 to 3 days if the symptoms continue.^1^

A task force set up by the European Paediatric Association concluded that the overconsumption of antibiotics in children with spontaneously recovering respiratory tract infections is 1 of the 4 most important health care problems in children in Europe.^3^ Indeed, many children with signs and symptoms of OM during acute respiratory infection recover spontaneously, and a wait-and-see practice with delayed introduction of antibiotics, if needed, is justified in appropriate cases and eventually reduces the consumption of antibiotics at the population level.

The aim of the present study was to evaluate antibiotic prescriptions for children with OM in a nationwide private network of outpatient clinics in Finland. Special focus was paid to amoxicillin and amoxicillin-clavulanic acid prescriptions, which have been the first-choice recommendations since 2017, according to Finnish guidelines. A nationwide dataset allowed an estimation of variations by age, time, region and the specialty of the physicians. Further, we were interested in identifying potential needs and specific areas for future active interventions antibiotic use among children with OM.

## METHODS

The study was conducted using data obtained from the electronic health records (EHR) of Terveystalo, the largest private healthcare company in Finland, with approximately 250,000 pediatric visits annually around the country. Terveystalo has more than 300 clinics covering all 20 hospital districts in Finland.

**FIGURE 1.**
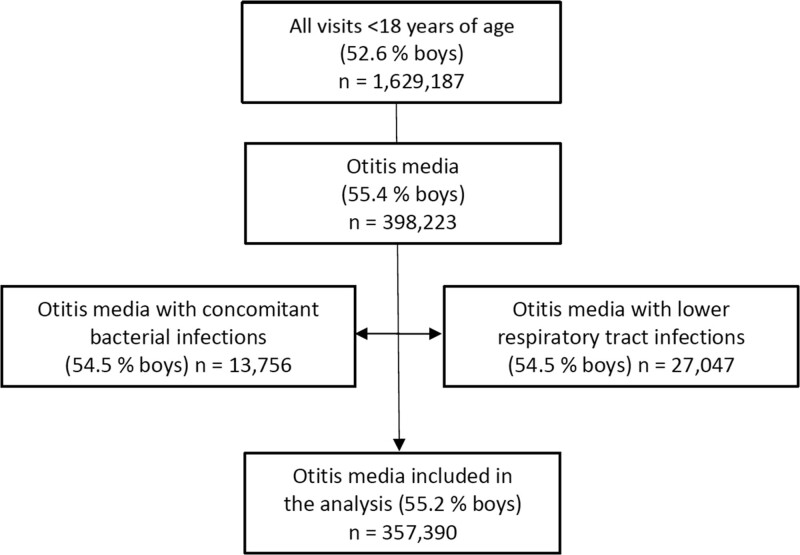
Flow of study inclusion and exclusion criteria for otitis media visits during the period of 2014–2020. Side arrows indicate the exclusion criteria. n, number of visits.

The study population consisted of children <18 years of age with an outpatient visit at Terveystalo from January 1, 2014, through December 31, 2020, who received an OM diagnosis [International Classification of Diseases (ICD) 10 codes H65, H66, H67]. The number of OM cases was 398,223 (55.4% boys) during the 7 surveillance years (Fig. 1). Patients with OM and concomitant bacterial infections, such as streptococcal pharyngitis, bacterial sinusitis, urinary tract or skin infections, were excluded. Patients with lower respiratory tract infections were also excluded. The Finnish Current Care guidelines recommend antibiotics for children with pneumonia,^4^ and in the same study population from which the present cases were selected, uncomplicated acute bronchitis was treated with antibiotics in 30% of the cases.^5^ The rationale beyond the exclusion was that a lower respiratory tract infection, if present, would influence the start and selection of antibiotics. Overall, 357,390 OM cases (55.2% boys) were included in the present study.

Dynamic Health (TietoEVRY, Finland) is a tool utilized by Terveystalo for handling EHR data, as described previously.^6^ In short, the data for this study were collected from EHR records containing visit information, coded diagnoses, and prescribed medications, as registered online by the practitioner in Dynamic Health. Most codes, such as the ICD-10, are mandatory in EHRs. Prescribed antibiotics were identified using the Anatomical Therapeutic Chemical Classification.

The patient’s birth date was used to calculate the actual age at the visit in 2014–2020. Children with OM were classified into 4 age groups: <2 years, 2–4.9 years, 5–11.9 years and 12–17.9 years. The physicians were classified into pediatricians, general practitioners (GP), ear, nose and throat physicians (ENT) and others. The years 2018–2020 represented the era after the publication of the updated guidelines in 2017.

### Statistics

All 357,390 OM visits during the study period were included in the analyses. IBM SPSS Statistics for Windows, version 26 (IBM Corp., NY), was used for the data analysis. Basic univariate analyses were conducted using the χ^2^ test. The 95% confidence intervals (95% CI) for the proportions were calculated using the Wald method (Stata 16.1 TX). Age and gender of the child, specialty of the physician and visit year were included in multivariate logistic regression, and the results were expressed as adjusted odds ratios (OR) and 95% CIs. The results were mostly presented as proportional rates to take into account the fact that respiratory infections were reduced by lockdown measures because of the COVID-19 pandemic in 2020.^7,8^

### Ethics

This study was a quality assessment and development project. All data were coded, and the patients were not contacted. All patient data were actively managed and conformed to the European Union’s General Data Protection Regulation and the data security legislation of Finland. According to Finnish law, approval from the Ethics Committee was not required, and informed consent was not needed from the subjects and/or their guardians. This study was carried out in accordance with national regulations and approved by the Chief Medical Officer of Terveystalo.

## RESULTS

The annual number of OM visits was highest in 2017 (62,450) and lowest in 2020 (26,252) (Table SDC1, Supplemental Digital Content 1, http://links.lww.com/INF/E813). The proportion of OM visits from all visits varied between 21.5 and 24.4% in 2014–2019, being substantially lower, 15.2%, in 2020 (see Table, Supplemental Digital Content 1, http://links.lww.com/INF/E813).

Antibiotics were prescribed to 44.8% of all OM patients; the in-group rate was lowest (44.1%, 51,082/115,836) in <2 years old and highest (46.8%, 41,847/89,488) in children 5–11.9 years old. GPs prescribed antibiotics to 54.0% (68,657/127,124), ENT physicians to 39.8% (39,979/100,346) and pediatricians to 38.8% (46,142/118,777) of children with OM (Table SDC2, Supplemental Digital Content 2, http://links.lww.com/INF/E813).

There were 27,047 children with OM associated with lower respiratory tract infections, and these cases were not included in the study. We compared the basic data between this excluded group and the study group, and children <2 years of age, boys, and visits to pediatricians were over-represented in the excluded cases (see Table, Supplemental Digital Content 2, http://links.lww.com/INF/E813).

**FIGURE 2.**
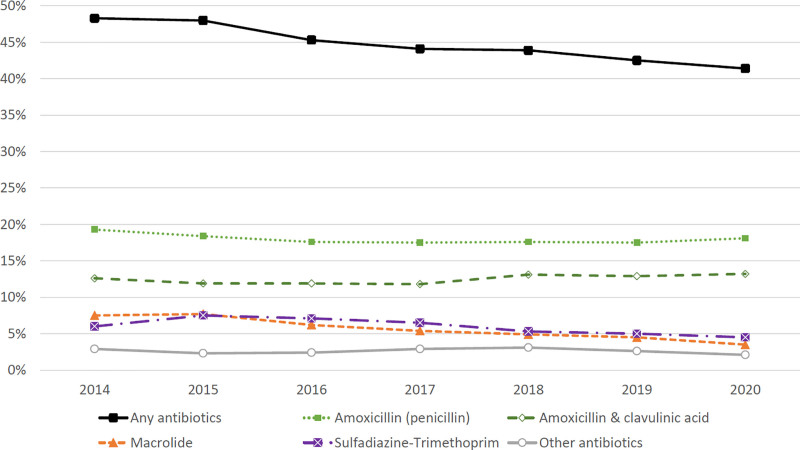
Antibiotic prescription rates for acute otitis media (n = 357,390).

The overall prescription rate of antibiotics for OM decreased from 48.3% in 2014 to 41.4% in 2020 (Fig. 2). The rates were 19.3% and 18.1% for amoxicillin, 12.6% and 13.2% for amoxicillin-clavulanic acid and 7.5% and 3.5% for macrolides, respectively (Fig. 2). Thus, the use of macrolides decreased by half during the 7 surveillance years. A minor increase in amoxicillin-clavulanic acid prescriptions took place from 2017 onwards. There were substantial variations in antibiotic prescriptions among clinics across the country, as presented by the differences between the hospital districts (see Figure, Supplemental Digital Content 3, http://links.lww.com/INF/E813).

When only patients who were treated with antibiotics were included, amoxicillin was the most often prescribed drug in all age groups (37.6–44.3%), followed by amoxicillin-clavulanic acid (23.9–29.8%) and macrolides (11.9–13.9%). Sulfa-trimethoprim was prescribed in 16.1% of children <2 years of age, and thereafter, the rate consistently decreased by age, being 6.0% in 12–17.9-year-olds (see Figure, Supplemental Digital Content 4, http://links.lww.com/INF/E813).

**FIGURE 3.**
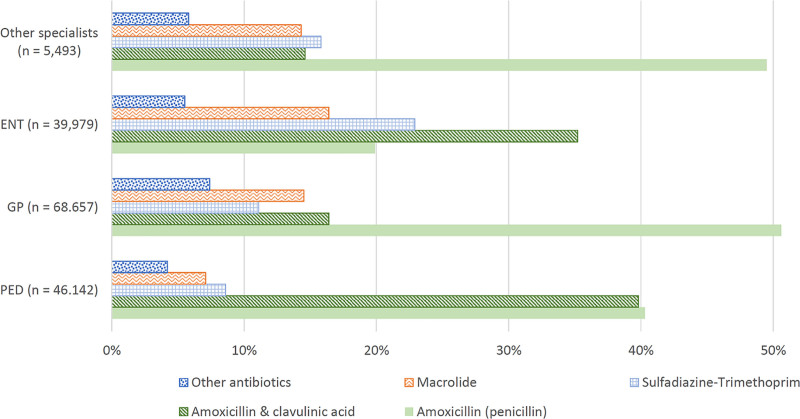
Children with otitis media who were prescribed antibiotics by different specialists. ENT, ear, nose, and throat specialist; GP, general practitioner; PED, pediatrician. (n = 160,271).

**FIGURE 4.**
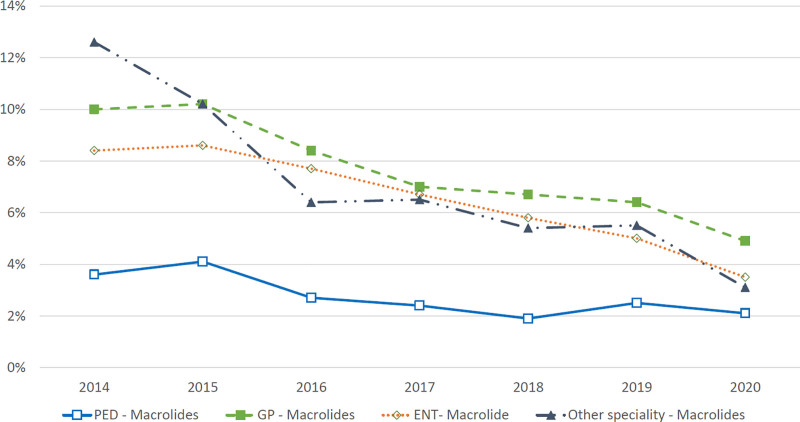
Macrolide prescription rate by physician’s specialty for otitis media among those who received antibiotic treatment. ENT, ear, nose, and throat specialist; GP, general practitioner; PED, pediatrician. (n = 160,271).

During the 7-year surveillance period, pediatricians prescribed amoxicillin to 40.3% and amoxicillin-clavulanic acid to 39.8% of children with OM (Fig. 3). The corresponding proportions for GPs were 50.6% and 16.4%, and for ENT physicians, they were 19.9% and 35.2%, respectively (Fig. 3). The prescribing of other drugs also varied significantly by specialty (Fig. 3). Macrolide prescriptions by GPs and ENT physicians halved between 2014 and 2020 (Fig. 4). Pediatricians prescribed macrolides substantially less often in all study years; therefore, the decrease, although clearly present, was milder (Fig. 4).

### Guideline concordance

The guideline concordance was evaluated by including data from the years 2018 to 2020 after the release of the updated guidelines. The concordance, defined as the prescription of amoxicillin or amoxicillin-clavulanic acid, was 81.1% (12,469/15,373) for pediatricians, 69.7% (14,782/21,193) for GPs and 60.4% (8,177/13,537) for ENT physicians.

The results on guideline concordance were confirmed by including the age and sex of the child, the visit year, and the specialty of the physician, in addition to the concordance of the prescription (yes/no) in multivariate logistic regression (Table 1). The results showed that the age, visit year and specialty of the physician were significantly associated with guideline concordance. The adjusted OR was 1.01 for age and 1.15 for visit year, and compared to pediatricians, the adjusted OR was 0.53 for visits to GPs and 0.35 for ENT physicians. This means that the risk of receiving a prescription that is not a guideline-concordant was 1.89-fold higher when visiting GPs and 2.86-fold higher when visiting ENT physicians when compared to pediatricians.

**TABLE 1.** Logistic regression analysis concordant antibiotics (amoxicillin with or without clavulanic acid) during the years 2018–2020 (after the release of updated guidelines)

		95% CI for OR		
	OR	Lower	Upper	*P* value
Gender (boy)	1.100	1.062	1.140	<0.0001
Age	1.010	1.005	1.015	<0.0001
Visit year	1.149	1.122	1.178	<0.0001
Speciality				
Paediatrician	1.000			
General practitioner	0.531	0.506	0.557	<0.0001
Earn nose and throat specialist	0.350	0.333	0.368	<0.0001
Other speciality	0.480	0.436	0.529	<0.0001

CI, confidence interval; OR, odds ratio.

We performed an *ad hoc* sensitivity analysis by excluding the 2020 data from the model to eliminate the effects of the COVID-19 pandemic. The results remained similar, with ORs being 1.01 (95% CI: 1.01–1.02) for age, 1.06 (1.02–1.10) for year, 0.52 (0.50–0.55) for visit to GP, and 0.35 (0.33–0.37) for ENT physicians.

## DISCUSSION

There are 3 main results in this real-life, 7-year observational study on antibiotic prescriptions for over 350,00 children with OM in private primary care. First, there was a substantial decrease in macrolide prescriptions, which led to a decrease in overall antibiotic prescriptions from 48.3% of children with OM in 2014 to 41.4% in 2020. Second, antibiotic choices varied significantly according to the doctor’s specialty. When only patients who were treated with antibiotics were included in the analyses, pediatricians prescribed amoxicillin or amoxicillin-clavulanic acid to 80% of OM patients, compared to 67% by GPs and 55% by ENT physicians. Amoxicillin was chosen most frequently by GPs, and macrolides by ENT physicians. Pediatricians chose amoxicillin and amoxicillin-clavulanic acid equally often. Third, during the postguideline years of 2018–2020, concordance with recommended OM treatment was highest among pediatricians and lowest among ENT physicians.

The 2011 Finnish Current Care Guidelines recommended amoxicillin as the first choice and amoxicillin-clavulanic acid as an alternative second-choice antibiotic for acute OM, and the 2017 updated guidelines recommended both amoxicillin and amoxicillin-clavulanic acid as first-choice drugs.^1^ This led to a minor increase in amoxicillin-clavulanic acid prescriptions. If the finding in otoscopy suggests nonpurulent OM, both guidelines recommend refraining from antibiotics and re-examination after 2–3 days if symptoms continue. This so-called wait-and-see practice has led to reduced antibiotic prescriptions and has been cost-effective compared to usual care.^9^

In our study, there was a relative decrease of 14.3% in the overall prescriptions of antibiotics between 2014 and 2020. This suggests that, in 2020, over half of the patients did not receive antibiotics. Our study design did not allow us to assess the exact reasons for this observed reduction, such as a possible increase in wait-and-see practice.^9^ Similar decreasing trends in antibiotic prescriptions have been found in other studies.^9^ A probable explanation is the gradual adaptation to widely disseminated local clinical practice or current care guidelines. A systematic review on the impact of OM treatment guidelines in children included 7 observational studies conducted in 6 countries.^10^ Six studies primarily recommended analgesics and the wait-and-see practice.^11–16^ In 5 studies that reported antibiotic prescription rates,^11–15^ 3 showed a decline of 5%–12% up to 3 years after guideline introduction,^11,13,14^ but 2 found no effects.^12,15^

The decrease in antibiotic use for OM is desirable, and in certain settings, the wait-and-see approach can be carried out without significant risks of complications.^17^ However, rechecking the ears by a physician within a few days is not always feasible, and in this case, more children with OM may receive immediate antibiotic therapy. Nevertheless, young children and those with purulent OM with severe symptoms require immediate antibiotics.^18^ In the end, it is challenging to determine the level of antibiotic use that is warranted and safe in a particular population. For this reason, the correct selection of an antibiotic is essential.

The abovementioned systematic review of 7 studies^10^ showed that the effects of clinical practice guidelines on antibiotic prescribing for acute OM were modest at most. The first-choice antibiotics were prescribed 9%–58% more frequently after guideline introduction in 4 studies.^11,14,16,19^ Similarly, statistically significant but clinically small to modest improvements in guideline adherence were reported in large register-based studies from Korea and the US after the release of the guidelines.^20,21^

Our results indicate that the overall reduction in antibiotic prescriptions was mainly due to the reduction in macrolide use. This was an advantageous change since macrolides poorly penetrate the middle ear and only narrowly affect the range of bacteria in acute OM.^22^ In a meta-analysis, macrolides were effective against 65%, amoxicillin against 85% and amoxicillin-clavulanic acid against 95% of bacteria cultured from middle ear secretions.^23^

In the present study, visiting a pediatrician was associated with a higher likelihood, and visiting a GP or an ENT physician was associated with a lower likelihood of receiving guideline-concordant antibiotics. Similar differences in antibiotic prescription habits favoring pediatricians have also been reported earlier.^24–26^ It would be reasonable to assume that the doctor’s specialty could influence the accuracy of OM diagnosis. This is a factor that could impact the rate of antibiotic use but not the choice of an antibiotic. It seems that the treatment decision is based on established habits, and perhaps guidelines related to the pediatric population are more readily adopted by pediatricians than by physicians who treat a wide range of age groups.

The COVID-19 pandemic started at the end of 2019 and was ongoing during the last year of our study. Common experience has been that, because of social distancing, restrictions and hygiene enhancements, the circulation of all respiratory viruses were lower during the pandemic than at respective times in previous years.^7,8^ In the present study, the number of OM visits in 2020 was less than half of that in 2018–2019, and the proportion of OM cases to all pediatric visits decreased by one-third. Since the data were mostly presented as ratios, it is unlikely that the change in visit numbers had a significant effect on the results of antibiotic prescriptions or guideline concordance.

The main strength of the present study was that the analyzed data came from outpatient clinics covering different areas across the country and consisted of more than 350,000 OM cases in <18 years old children. The information was electronically registered by the physician and was obtained for this study from a centralized and uniformly coded EHR system. Nevertheless, our study has some limitations. The data were retrospective and came from the private sector and thus may not fully represent the whole child population, although the difference between the public and private sectors may not be outstanding when acute infections in children are concerned. For example, the annual number of now-included cases was about 40% of those reported from public healthcare centers in Finland. Our design did not allow us to separate primary visits from revisits or controls after OM treatment. However, routine control visits after OM are currently against the guidelines. Neither was it possible to evaluate whether the OM diagnoses were correct. The assessment of guideline concordance was possible for the selection of antibiotics but not for whether there was under- or over-treatment with antibiotics.

## CONCLUSION

We found evidence that adherence to the Finnish Current Care guidelines on the treatment of OM in children was rather good. Less than half received antibiotics, and the great majority received amoxicillin or amoxicillin-clavulanic acid, which were the first-choice recommendations in the guidelines. To further increase concordance, active interventions should especially be targeted to tackle the identified challenges (eg, specialty or age group). Existing EHR systems could be armed with automated guideline-specific algorithms tailored according to diagnosis, patient group and physician’s specialty. Combining such systems with real-time monitoring and systematic feedback for physicians could facilitate the adaptation of various guidelines to day-to-day practice.

## Supplementary Material


