# Supplementary material for: Outpatient Antibiotic Prescribing for 357,390 Children With Otitis Media
Source: Pediatr Infect Dis J. 2022 Sep 7;41(12):947–52. doi: 10.1097/INF.0000000000003693 (PMC9645548; doi:10.1097/INF.0000000000003693)

**Supplement Digital Content**

**SDC 1.** Annual number of otitis media (OM) visits and the ratio of OM visits to all visits.

| **Year** | Number of OM visits | Ratio of OM  visits to all visits |
| --- | --- | --- |
| 2014  2015  2016  2017  2018  2019  2020 | 46,202  47,603  54,588  62,450  60,108  60,187  26,252 | 21.5 % 21.9 % 22.8 % 24.4 % 22.8 % 22.8 % 15.2 % |

**SDC 2.** Description of the study population and the patients excluded due to concomitant lower respiratory tract infection warranting antibiotic treatment. OM, otitis media

|  | **Study population** | | **Excluded patients** |
| --- | --- | --- | --- |
|  | **All OM included in the study, number (%)** | **Antibiotics prescribed for OM, number (%)** | **OM with LRTI excluded from the study, number** |
| All | 357,390 | 160,271 (44,8) | 27,047 |
| **Age (years)** |  |  |  |
| < 2 | 115,836 | 51,082 (44.1) | 11,667 |
| 2-4.9 | 132,705 | 58,765 (44.2) | 9,765 |
| 5-11.9 | 89,488 | 41,847 (46.8) | 4,651 |
| 12-17.9 | 19,361 | 8,577 (44.3) | 964 |
|  |  |  |  |
| **Sex** |  |  |  |
| Boy | 197,136 (55.2) | 87,459 (54.7) | 16,065 |
|  |  |  |  |
| **Speciality** |  |  |  |
| PED | 118,777 | 46,142 (38.8) | 16,361 |
| GP | 127,124 | 68,657 (54.0) | 61,92 |
| ENT | 100,346 | 39,979 (39.8) | 1,834 |
| Others | 11,143 | 5,493 (49.3) | 2,660 |

P <0.0001 for the difference between children with and without antibiotics prescribed, in relation to age, sex and speciality, respectively (Chi-squared test)
P <0.0001 for the difference between OM group included the study and OM with LRTI excluded from the study, in relation to age, sex and speciality, respectively (Chi-squared test)

**SDC 3.** Antibiotic prescription rates for acute otitis media in different hospital districts with more than 10,000 visits (n = 357,390)


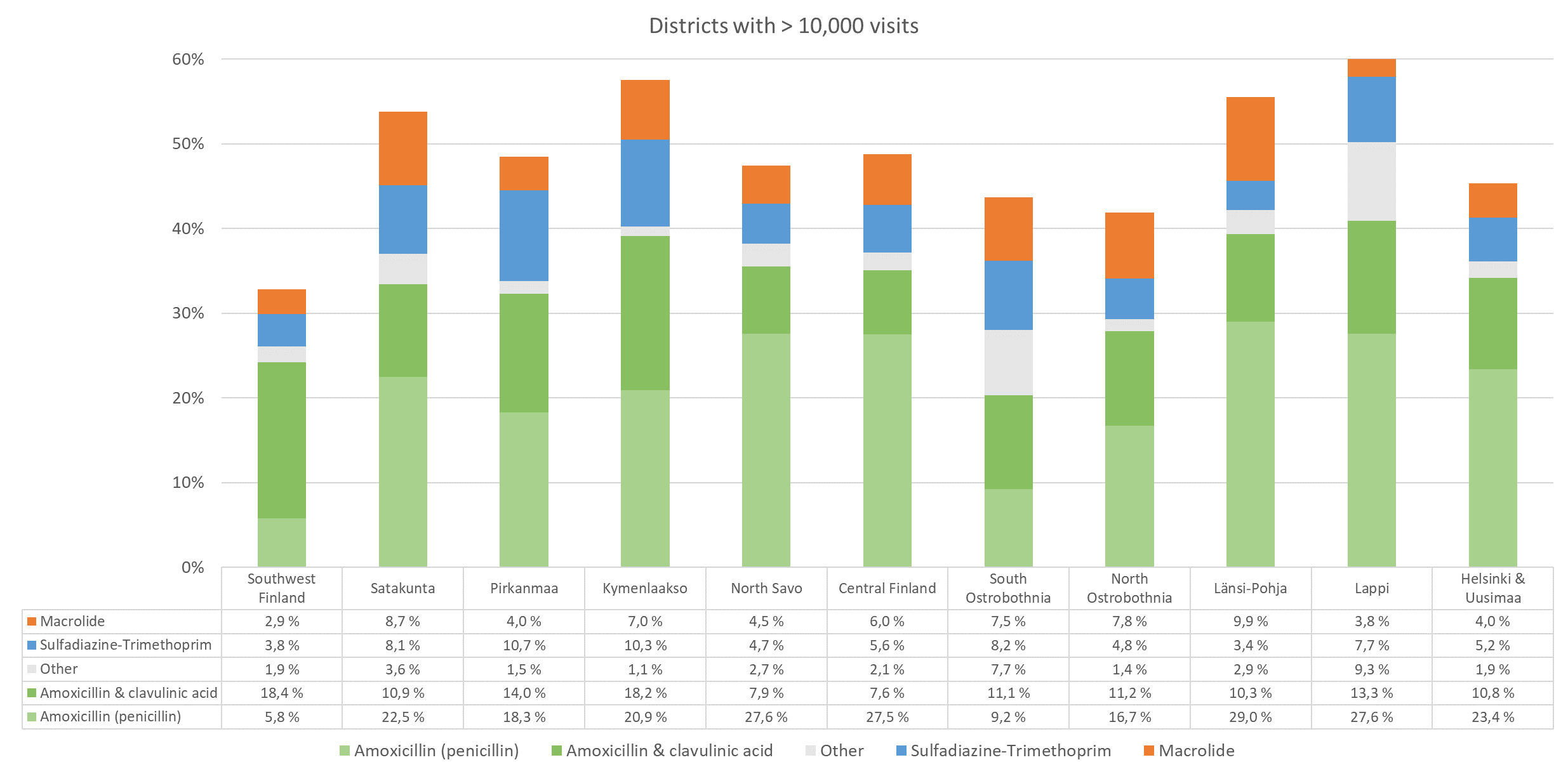


**SDC 4.** Children with OM who were prescribed antibiotics by age groups (n = 160,271)


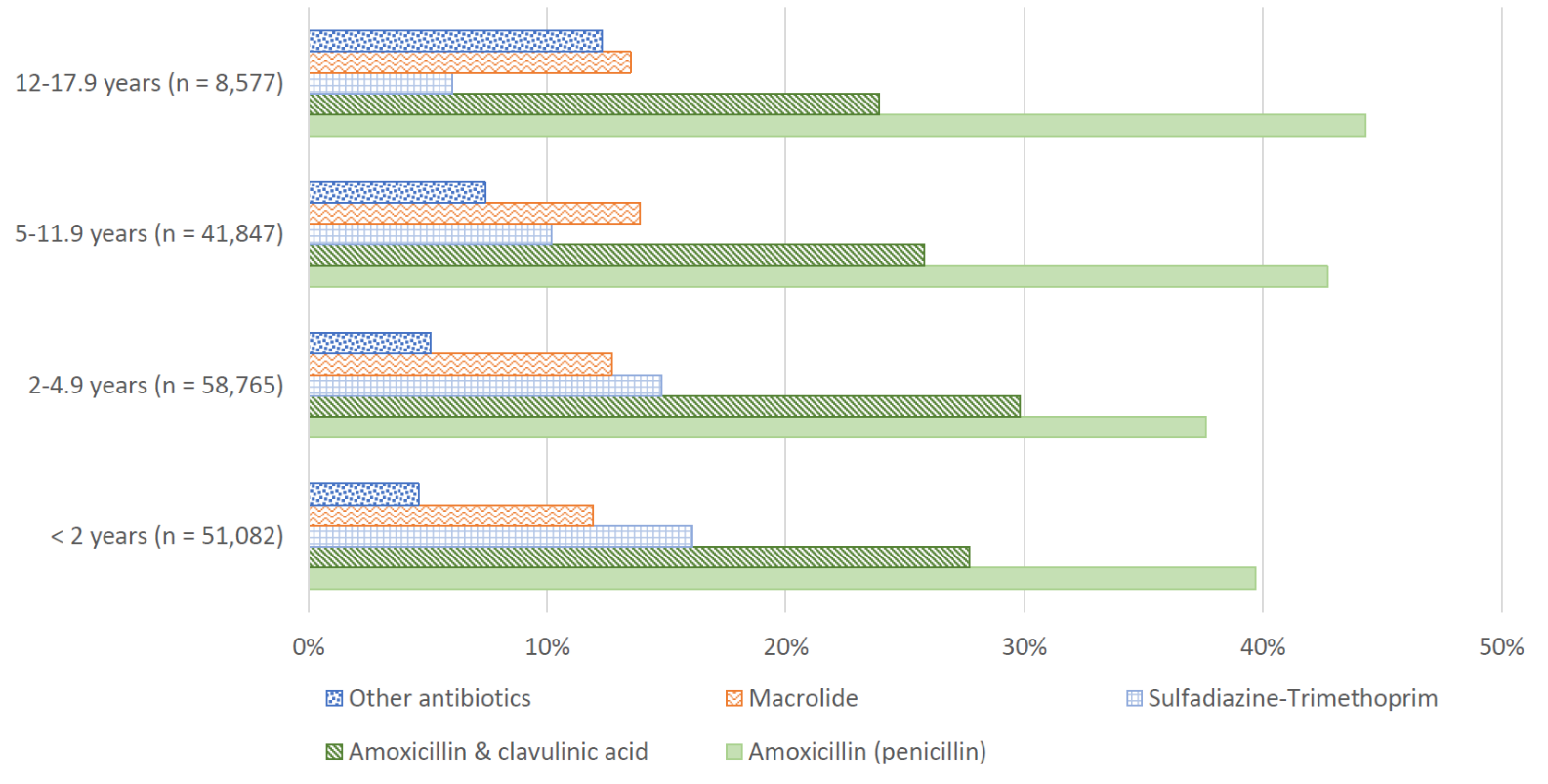

Supplement: Supplementary file 1 [file inf-41-0947-s001.docx]
